# Three Groups of Transposable Elements with Contrasting Copy Number Dynamics and Host Responses in the Maize (*Zea mays* ssp. *mays*) Genome

**DOI:** 10.1371/journal.pgen.1004298

**Published:** 2014-04-17

**Authors:** Concepcion M. Diez, Esteban Meca, Maud I. Tenaillon, Brandon S. Gaut

**Affiliations:** 1 Dept. of Ecology and Evolutionary Biology, UC Irvine, Irvine, California, United States of America; 2 Departamento de Agronomía, Universidad de Córdoba, Campus de Excelencia Internacional Agroalimentario, ceiA3, Cordoba, Spain; 3 Department of Mathematics, UC Irvine, Irvine, California, United States of America; 4 CNRS, UMR de Génétique Végétale, INRA/CNRS/Univ Paris-Sud/AgroParisTech, Ferme du Moulon, Gif-sur-Yvette, France; Harvard University, United States of America

## Abstract

Most angiosperm nuclear DNA is repetitive and derived from silenced transposable elements (TEs). TE silencing requires substantial resources from the plant host, including the production of small interfering RNAs (siRNAs). Thus, the interaction between TEs and siRNAs is a critical aspect of both the function and the evolution of plant genomes. Yet the co-evolutionary dynamics between these two entities remain poorly characterized. Here we studied the organization of TEs within the maize (*Zea mays* ssp *mays*) genome, documenting that TEs fall within three groups based on the class and copy numbers. These groups included DNA elements, low copy RNA elements and higher copy RNA elements. The three groups varied statistically in characteristics that included length, location, age, siRNA expression and 24∶22 nucleotide (nt) siRNA targeting ratios. In addition, the low copy retroelements encompassed a set of TEs that had previously been shown to decrease expression within a 24 nt siRNA biogenesis mutant (*mop1*). To investigate the evolutionary dynamics of the three groups, we estimated their abundance in two landraces, one with a genome similar in size to that of the maize reference and the other with a 30% larger genome. For all three accessions, we assessed TE abundance as well as 22 nt and 24 nt siRNA content within leaves. The high copy number retroelements are under targeted similarly by siRNAs among accessions, appear to be born of a rapid bust of activity, and may be currently transpositionally dead or limited. In contrast, the lower copy number group of retrolements are targeted more dynamically and have had a long and ongoing history of transposition in the maize genome.

## Author Summary

Because transposable elements (TEs) constitute most angiosperm nuclear DNA, the interaction between TEs and their host genome is a key component for understanding the function and evolution of plant genomes. The diversity of the host response has been studied a great deal, including the biogenesis of small interfering RNAs (siRNAs) that target TEs for epigenetic modifications. However, little is known about variation in TE content among closely related genomes and whether siRNA expression tracks this variation. To that end, we surveyed both the copy number and the siRNA targeting of more than 1500 distinct TE subfamilies in the B73 maize reference genome. These surveys indicated that TE subfamilies fall naturally into three distinctive groups based on their class and copy number, but these groups also differ with respect to their location in the genome, their age, their expression and their siRNA regulation. The presence and consistency of these TE groups was also assessed in two genetically distant maize landraces with contrasting genome sizes. The variation in siRNA targeting across different TE groups and families, as well as the lack of correlation between TE and siRNA abundances, argues for the existence of multiple mechanisms and strategies for TE silencing.

## Introduction

Most DNA within angiosperm genomes is repetitive, typically representing active transposable elements (TEs) or DNA derived from formerly active TEs. This repetitive component is the primary determinant of genome size (GS) variation across species, constituting ∼20% of small genome species like rice and *A. thaliana* but >85% of larger genomes like that of maize (*Zea mays* ssp. *mays*), barley and wheat [1]. The preponderance of TE-derived DNA suggests superficially that TEs reign unchecked within plant genomes, but this is of course untrue because natural selection acts both to attenuate TE activity and to remove them from genomes and populations [1], [2].

TE activity is also attenuated by the plant host, which uses small interfering RNAs (siRNAs) to silence TEs both before and after transcription. Many of the molecular details of this host response remain unclear, but the general mechanism of pre-transcriptional silencing is now well known [3]–[5]. TEs are first recognized by the host, probably via double-stranded RNAs that originate either as a consequence of a hairpin structure in the RNA or by complementary transcripts from different strands. These double-stranded RNAs are cleaved by DICER complexes into 24 nucleotide (nt) fragments, and the 24 nt siRNAs are loaded onto an Argonaut complex, which migrates to a precise chromosomal location based on homology between the DNA-target and the 24 nt siRNA. The Argonaut complex then attracts methylation machinery, leading to *de novo* TE methylation and silencing.

Post-transcriptional silencing is not as thoroughly characterized, but it appears to rely primarily on siRNAs of 21 nt in length for most plants but predominantly of 22 nt in length for maize (*Zea mays* ssp. *mays*) [5], [6]. The 21/22 nt siRNAs may originate by several mechanisms, including from miRNA genes, from phased processing of RNAs [7] and from digestion and processing of mRNAs [8], [9]. No matter the source, 21/22 nt siRNAs target mRNA transcripts through homology, with the consequent double-stranded RNA either modified or degraded [3], [5].

Ultimately the host response leads to the attenuation of TE activity and limits TE copy number. However, TEs may occasionally escape host control, leading to a ‘burst’ of transposition, an increase in copy number and potentially a shift in genome size [10], [11]. Although not well characterized, bursts of activity may vary by TE type, for at least two reasons. First, TEs have inherently different multiplication capabilities [12]. Cut-and paste class II DNA transposons replicate conservatively, while copy-and-paste class I retroelements have the capability to replicate multiplicatively. Second, the host response can vary with the TE subfamily [13], [14]. This variation in host response has become obvious in part from the study of methylation mutants. For example, mutants with modified activity of RNA-dependent RNA polymerase 2 (RDR2) produce fewer 24 nt siRNAs than wild type, with a concomitant increase in TE transcription [7], [13], [15]. However, in the maize RDR2 (*mop1*) mutant, TE transcription is actually *decreased* for a subset of TE subfamilies [13], illustrating that not all TEs are equal with respect to the mechanisms of the host response.

Despite the fact that the interaction between TEs and siRNAs is a critical aspect of genome function and evolution, the co-evolutionary dynamics between these two entities remains poorly characterized. Such characterization requires the study of covariation between siRNA expression and TE copy number. However, the estimation of TE copy numbers is not trivial because “complete” genomes often lack components of repetitive DNA. For example, the maize reference sequence is estimated to be missing ∼11% of the genome [16], most of which is likely to be repetitive elements. To get around this problem, Tenaillon et al. [17] have developed a method to estimate the TE complement in the maize genome based on high throughput sequencing (HTS) of genomic samples. In this method, the HTS reads are mapped against an exemplar set of sequences that represent ∼1500 TE subfamilies in the maize B73 reference genome [16]. By assessing the coverage of each exemplar, researchers have been able to not only to estimate relative contribution of individual TE subfamilies but also to identify some of the repetitive DNA that was missing from the reference [17], [18].

This study is born from an observation about TE abundance that is based on the data of Tenaillon et al. [17]. In perusing copy number among over ∼1500 TE subfamilies in the maize genome, we have noticed that TEs fall into three distinct groups based on their class and copy numbers. The first group is set of DNA (class II) transposons. Another is composed of high copy number retroelements, such as members of the *Opie* family of the Long Terminal Repeat (LTR) *Copia* superfamily and members of the *Cinful* family of the LTR *Gypsy* superfamily. The final group consists of over 300 retrolement subfamilies with lower copy number. This observation suggests that there is a higher-order organization of elements within the maize genome, and it has prompted us to study features of their evolutionary dynamics.

To characterize the groups, we first employ bioinformatic and genomic analyses of data from the B73 reference genome. Specifically, we have used newly generated siRNA data to compare and contrast patterns of the siRNA-mediated host response among TE groups. Then, to better understand the evolutionary dynamics of these groups, we compare TE abundances and siRNA profiles among B73 and two additional landraces, Palomero Toluqueño (PT) and Olote Colorado (OAXA). We have chosen these samples for two reasons. First, they are roughly equidistant in genetic relationship to the B73 reference; based on SNP data [19], the two landraces form an ingroup with B73 as the outgroup. The second reason is that they represent extremes of the ∼30% variation in genome size (GS) within the species [20]. PT has a genome size of 5.58 pg/2C, which is similar to that of the 5.64 pg/2C B73 reference genome, whereas the OAXA genome is ∼1.3-fold larger, at 7.11 pg/2C [20]. This extreme difference in GS enhances the *a priori* probability that there is, in fact, variation in TE copy numbers and siRNA expression in our sample of germplasm.

With genomic and siRNA HTS data from three accessions, we address a set of four questions. First, given that TEs fall naturally into three groups based on their class and copy numbers, do they vary in other characteristics? If so, what might these characteristics imply about genome organization and the host response? Second, are these three groups consistent across the maize germplasm, suggesting that this organization is a higher-order property of the maize pan-genome [21]? Third, do the groups vary in their evolutionary dynamics, as measured by differences in abundance among accessions? Finally, do shifts in siRNA expression covary with the abundance of the TEs they target? Our ultimate goal is to begin to unravel the evolutionary dynamics between TEs and the host response in the context of the history and organization of the maize genome.

## Results

### Copy number dynamics define three distinct groups of TEs within the genome

While surveying copy numbers of TEs within B73, we observed an interesting phenomenon. The observation began by mapping 18,689,555 paired-end (PE) reads of B73 genomic data to the published Unique TE (UTE) database. The UTE consisted of 1514 TEs that was built by filtering the exemplar database of 1526 TEs (TEdb) [16], [22] to reduce cross-homologies between TE exemplars and thereby improve mapping resolution [17].

**Figure 1:**
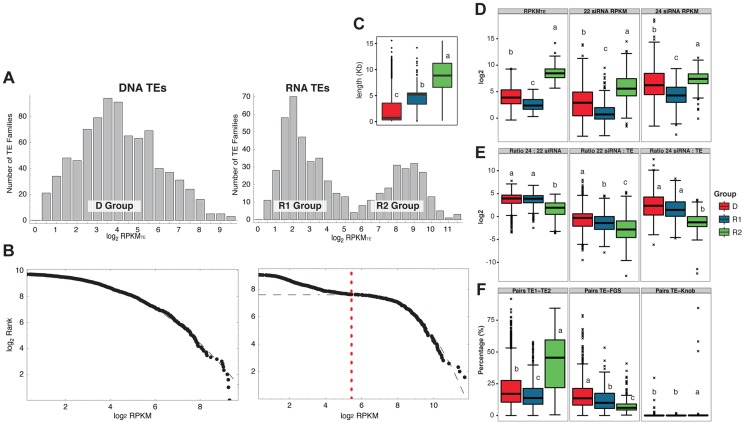
Characteristics of the three groups of TEs in B73, as defined by class and copy number. a) Histograms of the RPKM of TEs (left) and RNA elements, based on genomic reads. b) The empirical distribution function for DNA TEs (left) and RNA elements (right). The dots represent individual TE subfamilies and the dashed line is a fitted log-normal distribution. The vertical dashed red line is used to define groups R1 and R2. c) Lengths of the exemplar elements in the three groups. d) Characteristics of the three groups for TE, 22 nt siRNA and 24 nt siRNA abundances (RPKM values). e) Characteristics of the three TE groups for the 24 : 22 nt siRNA ratio (left) and a proxy for the number of 22 nt and 24 nt siRNA hits per TE copy (siRNA RPKM : RPKM_TE_). f) Graphs about the location of TEs based on paired reads: left, the percentage of paired reads in which both reads map to different TE exemplars of the UTE; middle, the proportion of paired reads in which one read maps to the UTE and the other to the FGS; right, the proportion of paired reads in which one of the reads maps to the UTE and the other to the KnobC database. For all boxplots in panels c, d, e and f, the boxes indicate the first quartile (bottom line), the median (central line) and the third quartile (upper line). The whiskers represent the highest and lowest values of the data that are within 1.5 times the interquartile range of the box edges. The outliers are represented by crosses. The lower case letters above the boxes represent significance groupings after a pairwise comparison. Boxplots sharing the same lower case letter are not significantly different at *p*<0.05.

Plots of the RPKM (Reads per Kilobase per Million mapped, see Methods) values for individual TE subfamilies (RPKM_TE_) yielded different distributions between DNA transposons and RNA transposons. The DNA transposons had a unimodal distribution of RPKM_TE_, while the RNA transposons had a bimodal distribution (Figure 1a). We constructed a Rank-Frequency plot, which is a representation of the Empirical Distribution Function (EDF), for these data and found that DNA (or class II) transposons closely matched a log-normal distribution (Figure 1b) but RNA elements did not. Instead, the RNA elements fit a mixture of a log-normal distribution and another (approximately Poisson) distribution. Based on these distributional properties, we defined three TE groups: group D, which consisted of 841 exemplar DNA elements; group R1, which included 365 exemplar RNA elements with relatively low abundances; and group R2, the set of 198 high abundance class I retroelements (Figure 1ab; Table S1). Note that these three groups do not include 110 exemplar elements for which the RPKM_TE_ data suggested fewer than 2 copies in B73.

Among the three groups, it may not be surprising that the ‘high copy’ R2 group contained retroelements known to be common throughout the maize genome, including *Ji* and *Opie Copia* elements and the *Cinful*, *Huck* and *Prem*1 *Gypsy* elements (Table 1) [22]–[24]. There is nonetheless substantial overlap in the identity of superfamilies between the R1 and R2 classes. For example, the R1 and R2 group include *Copia* (n = 95 and n = 52, respectively) and *Gypsy* (n = 128 and n = 112, respectively) exemplars, as well as a wide array of other LTR retroelements and LINE L1 elements (Table 1). Thus, at the gross levels of TE Order and Superfamily [25], there was extensive overlap between the R1 and R2 groups. Their primary distinction was abundance.

**Table 1:** Characteristics of TE families within the R1 and R2 groups.

Class Designation^1^	Number in Group R1	Number in Group R2	Description
RIT	0	2	LINE RTE
RLC_*Ji*	0	16	LTR *Copia*
RLC_*Opie*	0	17	LTR *Copia*
RLC (various)	95	19	LTR *Copia*
RLG_*Cinful*	0	41	LTR *Gypsy*
RLG_*Huck*	0	20	LTR *Gypsy*
RLG_*Prem1*	0	10	LTR *Gypsy*
RLG (various)	128	41	LTR *Gypsy*
RST	2	4	SINE tRNA
RLX	110	28	Unknown LTRs
RIL	30	0	Line L1
Total	365	198	

1 Designations and descriptions from [25]. TE families are listed when they consist of >10 subfamilies.

### The three groups have distinct genomic and historical properties

Given noticeable differences in abundance dynamics, we investigated additional characteristics among the three groups (Figure 1c–f) - including their genomic properties, siRNA targeting and insertion ages – to help determine whether the groups are differentiated by characteristics beyond abundance. We found that the abundant R2 group of retroelements was longer, on average, than the other two groups (Figure 1c), with the R1 group intermediate in length among the three. The groups also differed in genomic location (or context). We assessed genomic context by mapping paired-reads that did not match the same TE exemplar [17]. That is, if one paired-end matched a known TE exemplar, we could assess whether the second read matched to a second TE subfamily, to a gene in the Filtered Gene Set (FGS) or to a reference set of Knob and Centromeric (KnobC) repetitive DNA (see Methods). The results indicated that the D group was more often located close to genes [22], the R2 group was more often located near other TEs, and R1 elements were closer to genes on average than R2 elements (Figure 1f).

We assessed one aspect of the host response to these groups by sequencing 22 nt and 24 nt siRNA from B73 leaf tissue, resulting in a total of 9.23×10^6^ and 20.16×10^6^ reads, respectively, for the two size classes. These siRNA reads were mapped to the TEdb of 1526 elements [16], and we recorded the number of siRNA hits to each TE exemplar. The mapping results revealed that the R2 group had the highest total siRNA hits, in part due to their higher abundance (Figure 1d). However, when corrected for RPKM_TE_, these TEs tend to be lowly targeted by both 22 nt and 24 nt siRNAs on a per-copy basis (Figure 1e), perhaps because long retroelements are targeted primarily at their ends rather than across their entire length by siRNAs and methylation marks [26]–[28]. In contrast, the D and R1 TEs were targeted by significantly higher numbers of siRNAs per RPKM_TE_ and also by higher 24 : 22 nt siRNA ratios (Figure 1e).

**Figure 2:**
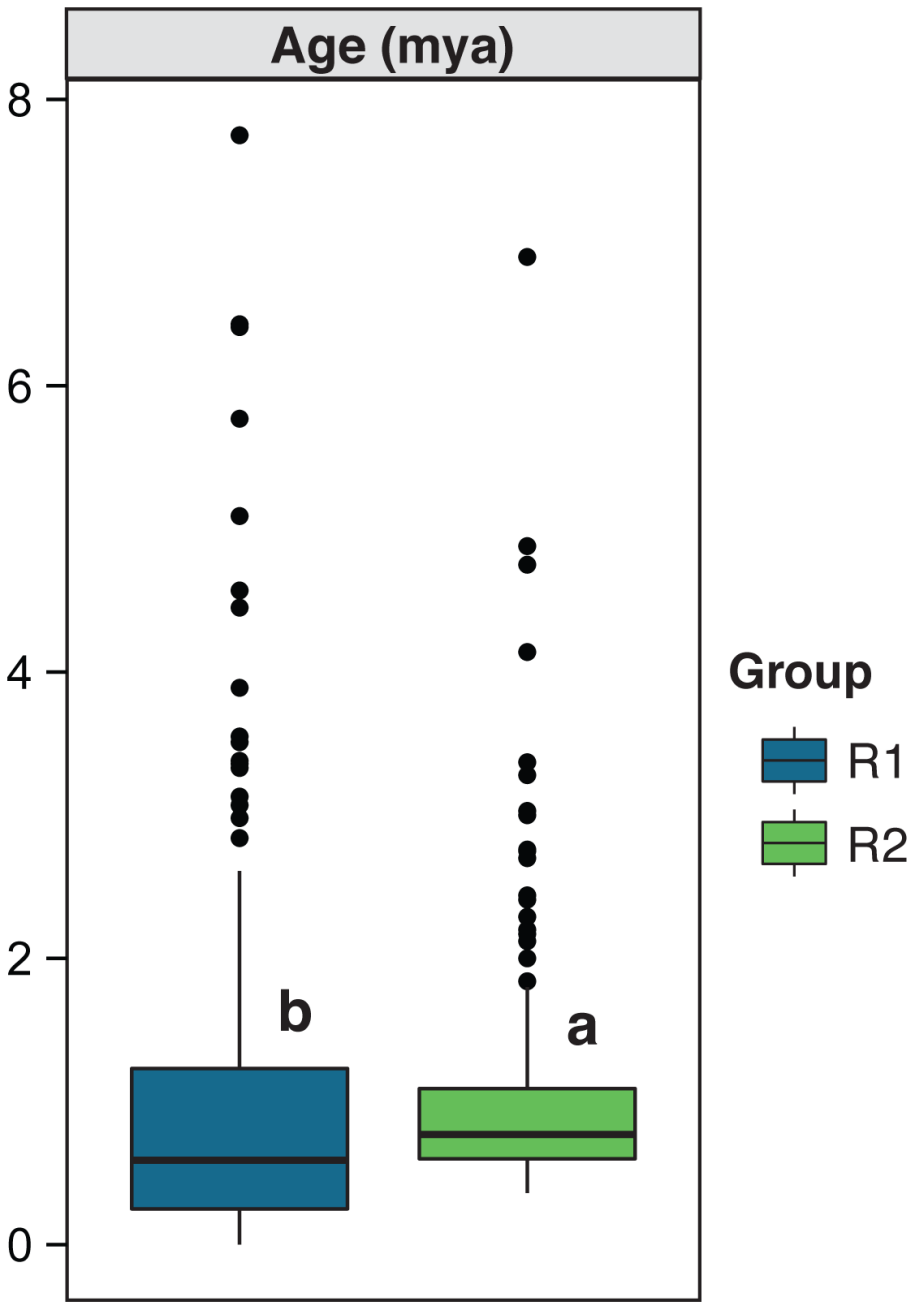
Age of the TE subfamilies included in groups R1 and R2 [22]. The boxes indicate the first quartile (bottom line), the median (central line) and the third quartile (upper line). The boxes, whiskers and dots for the boxplots are defined in the caption of Figure 1, as are the lower case letters above the boxes.

Finally, we summarized insertion time estimates of the R1 and R2 groups, using data from a previous study of the B73 genome [22] (Figure 2). Both groups exhibited heterogeneity in insertion times, with some elements estimated to be >5 million years (my) old. However, the average age of the two groups differed significantly (p<0.001, Kruskal-Wallis), with the R1 groups younger (average estimated age 0.93 my, n = 305, std. dev. 1.11) than the R2 group (average estimated age 1.04 my, n = 191, std. dev. 0.84). Moreover, the R1 group included elements with a range of insertion ages that included recent insertion (0.00 my). In contrast, the age distribution of the R2 group suggested that most element proliferation occurred in a well-defined period, with no evidence of insertion in the last 0.36 my.

To sum: While there is variation within the D, R1 and R2 groups for all measured characteristics (Figure 1), the three groups nonetheless differed significantly for most measured characteristics, including size, location, age and siRNA targeting. These differences suggest the three groups are biological entities with distinct properties.

### Expression of the R1 group is suppressed in *mop*1 mutants

**Figure 3:**
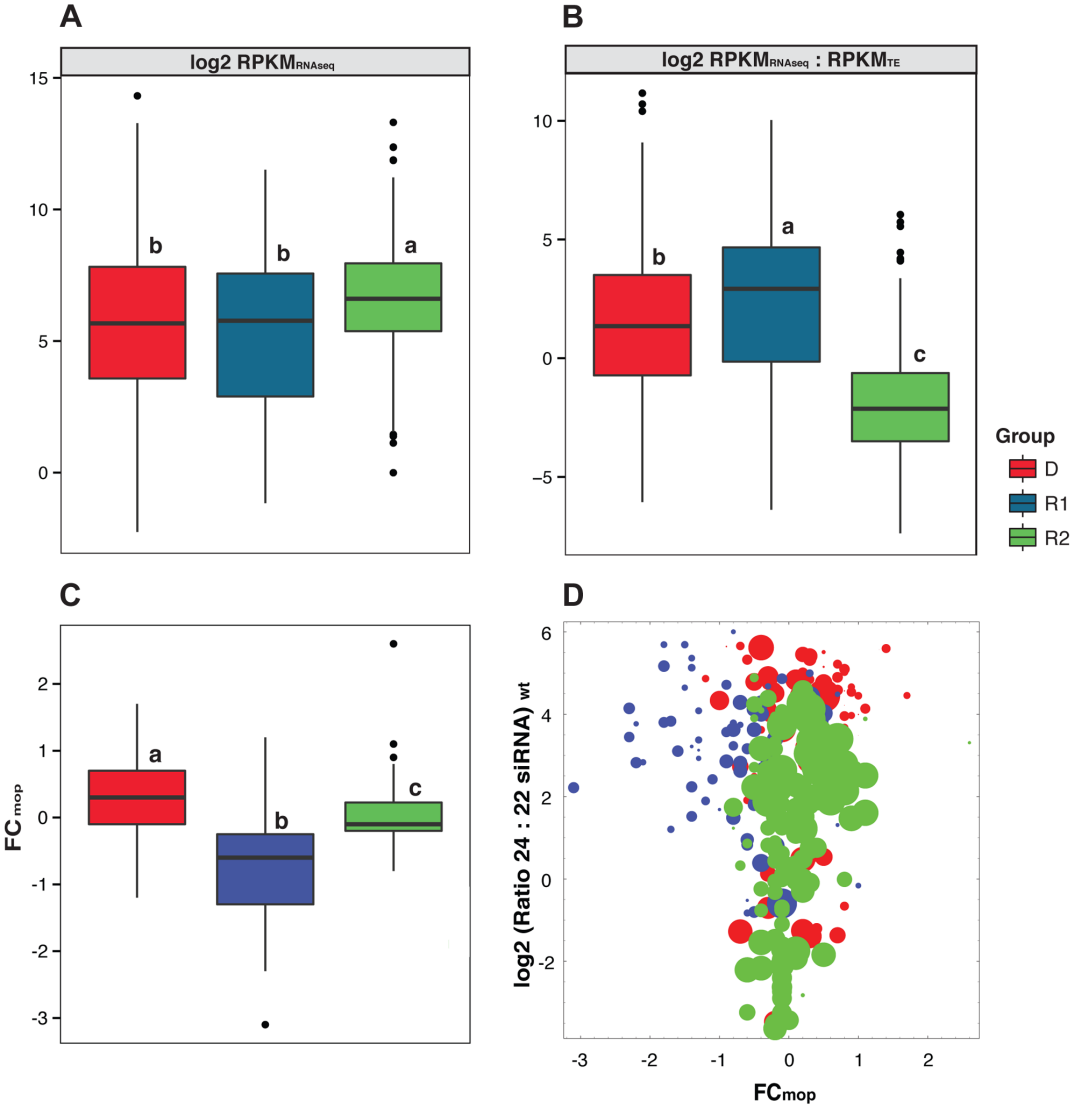
Expression characteristics of the three TE groups. a) Overall expression (RPKM_RNAseq_) and b) expression per TE copy (RPKM_RNAseq_ : RPKM_TE_) for the three TE groups based on RNAseq data from transition leaves [57] c) Fold-change in TE expression (FC_mop_) between wild type (*wt*) and the *mop1* mutant for a subset of 340 TEs [13]. d) A plot of FC_mop_ and the 24∶22 nt siRNAs ratio for the same 340 TE subfamilies (dots). The diameter of the dots is proportional to the length of the TE exemplar. The boxes, whiskers and dots for the boxplots are defined in the caption of Figure 1, as are the lower case letters above the boxes.

Given dramatic differences in age and siRNA targeting among groups, we also determined whether the groups differ in expression dynamics. To assess expression, we examined existing RNAseq data from B73 leaf tissue (see Methods). The data indicate that total expression of R2 elements is highest among the three groups, with similar levels of expression for the D and R1 groups (Figure 3a). However when corrected for abundance, the R2 TEs have the lowest expression on a per-copy basis (Figure 3b), consistent with the possibility of copy-number repression [29], [30]. In contrast, R1 elements exhibit the highest expression on a per-copy basis (Figure 3b). We found similar expression patterns based on germline (immature tassel) tissue (data not shown).

We also analyzed expression data to assess whether the three groups have different dynamics with respect to an interruption in the host response. To assess this phenomenon, we assessed RNAseq expression data from reference [13], which generated data from the shoot apical meristems of wild type (*wt*) and RDR2 *mop1* mutant plants in the W22 background. Jia *et al.* [13] reported 373 TE subfamilies with differential expression in the *mop1* mutant relative to the wild type (*wt*). Of these, we selected the 340 TE subfamilies with names that matched the exemplar TEs from the UTE (Table S2). [For this subset of 340 TEs, we first confirmed that the previous observations about length and other differences among groups continued to hold (Figure S1).] We then examined the fold-change (FC_mop_) in expression between *wt* and *mop1*. There were clear trends among groups. On average, expression of the D group was enhanced in the *mop*1 mutant; for the 109 TE subfamilies in the data set expression increased slightly, ∼0.29 log 2 units or ∼1.2-fold on average (Figure 3c). The 144 members of the R2 group in the dataset exhibited no strong tendency, with an average 1.03-fold shift in expression. In contrast, the R1 group experienced an average −1.6-fold *decrease* in expression in the *mop*1 mutant, with 80% (70 of 87) exemplars exhibiting a decrease. The effect of decreased expression was particularly prominent for TE exemplars targeted by high ratios of 24∶22 siRNA, based on our B73 leaf data (Figure 3d). Thus, the puzzling phenomenon of decreased TE expression in a maize RDR2 mutant is due to R1 elements.

### The three TE groups are evident in other maize genomes

We questioned whether the three TE groups were unique to the reference genome or a consistent genomic feature across maize *sensu lato*. To assess TE copy numbers across individuals, we sequenced one lane of genomic DNA from each of the landraces Palomero Toluqueño (PT) and Olote Colorado (OAXA). Recall that PT has a genome size of 5.58 pg/2C, which is similar to that of the 5.64 pg/2C B73 genome, whereas OAXA genome is 7.11 pg/2C [20]. Our Illumina sequencing yielded a total of 53,535,615 and 54,318,379 paired-end reads, respectively, for the two accessions (Table S1). These genomic HTS data were mapped to three databases: *i*) the Filtered Gene Set (FGS) [16], *ii*) the KnobC database and *iii*) the UTE. Briefly, the percentage of reads that mapped to the FGS and UTE was similar across accessions: 15.0% and 61.7%, respectively, for B73; 17.0% and 62.4% for PT; and 16.8% and 55.2% for OAXA. The largest difference between accessions was in the percentage of genomic HTS reads that mapped to the KnobC database (at 6.12% for B73, 1.26% for PT and 11.14% for OAXA). Thus, the most obvious difference between accessions was in heterochromatic sequences, consistent with previous studies suggesting that knob DNA is the primary determinant of GS differences within the genus *Zea* [18].

Given HTS data, we determined whether the R1 and R2 groups were consistent across accessions or simply a property of the B73 genome. We therefore calculated the RPKM_TE_ values based on reads from PT and OAXA (Figure S2). For both landraces, the retroelements had a bimodal distribution of copy number, consistent with the B73 analyses (Figure 1ab). Moreover, the same TE subfamilies fell within the two groups: across all three accessions, there was 97.3% agreement in classification to the R1 and R2 groups. Given this fact, we used the D, R1 and R2 groupings as defined in B73 for all ensuing analysis.

### Copy number dynamics among the groups

**Figure 4:**
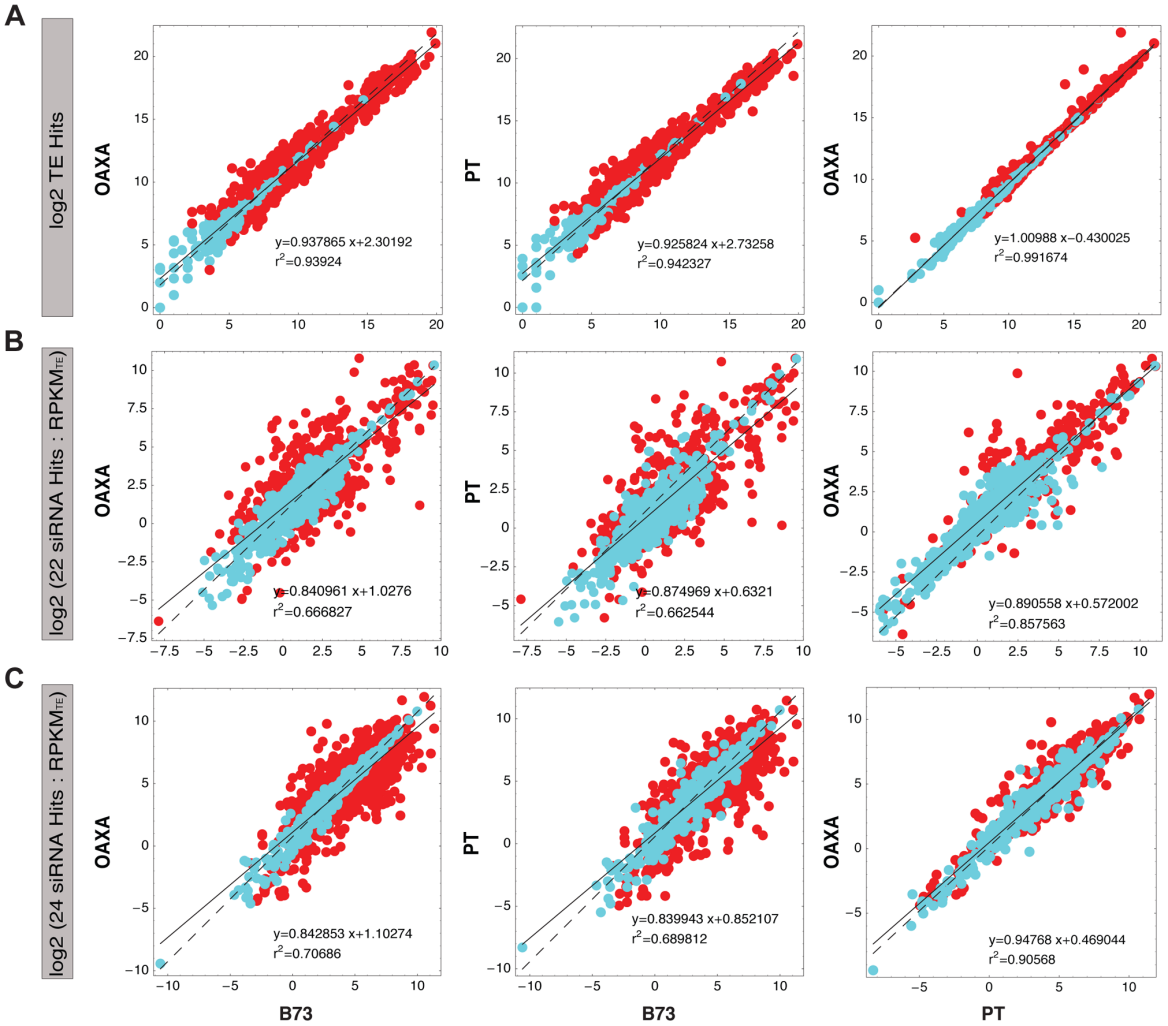
Pairwise comparisons between accessions for: a) TE hits; b) 22 nt siRNA hits per RPKM_TE_ and c) 24 nt siRNA hits per RPKM_TE_. For all the cases the *x*- and *y*-axis indicate accessions under comparison (B73, PT or OAXA). Each dot represents a TE subfamily, with the regression (*y*) and correlations (*r^2^*) between accessions indicated. The solid line represents the regression fit, while the dashed line represents the null hypothesis. The color of the dots represents significance: red dots are significant differences between accessions at a FDR of *q*<0.001, based on the χ^2^_Corr_ in panel a and the χ^2^_Prop_ for panels b and c. Blue dots are not significant.

Given the genomic data, we assessed whether the groups evolve similarly by focusing on shifts in abundance among accessions. We did this in two ways. First, for each of the 1514 TE exemplars in the UTE we assessed the number of mapped genomic reads to each exemplar; we then calculated correlations between accessions across all TE exemplars using a logarithmic transformation. The correlation in TE abundance was high for all three pairwise comparisons but highest for the PT and OAXA comparison (*r* ^2^ = 0.992 versus *r* ^2^ = 0.942 between PT and B73 and *r* ^2^ = 0.939 between B73 and OAXA) (Figure 4a). Despite these high pairwise correlations there were nonetheless detectable differences in TE abundances for individual TE subfamilies. We applied two statistical tests to assess linear differences between accessions based on the number of hits in each TE exemplar (Table 2). The first was a standard χ^2^ (χ^2^ *_Std_*) that compares the proportion of hits to a particular TE subfamily between two accessions; with a False Discovery Rate (FDR) of *q*<0.001, this method resulted in (for example) 834 TE subfamilies with detectable difference in abundance between PT and OAXA (Table 2). We also devised a novel χ^2^ (χ^2^ *_Corr_*) that corrects for the fact that different accessions may have different overall proportions of TEs within those genomes (see Methods). Based on this more appropriate method, 514 TE subfamilies (33%) differed between PT and OAXA, and ∼1000 TE subfamilies differed between B73 and each of the two landraces (Table 2). These results generated a ranked list of TE subfamilies that are most likely to vary between accessions (Table S1), but the results require further verification (see Discussion).

**Table 2:** The number of significantly differences in pairwise comparisons between genotypes for TE, 22

	TE abundance		22siRNA		24siRNA	
Pair	χ^2^ *_Std_* a	χ^2^ *_Corr_* b	χ^2^ *_Std_*	χ^2^ *_Prop_* c	χ^2^ *_Std_*	χ^2^ *_Prop_*
**B73-PT**	1029	1022	408	506	865	917
**B73-OAXA**	1001	1021	402	482	790	902
**OAXA-PT**	834	514	388	493	675	711

a The standard χ^2^ test based on a 2×2 table of the relative proportions of hits.

b The χ^2^ corrected by the coverage to the FGS.

c The χ^2^ test of proportionality; see text and Supplement Text S1.

**Figure 5:**
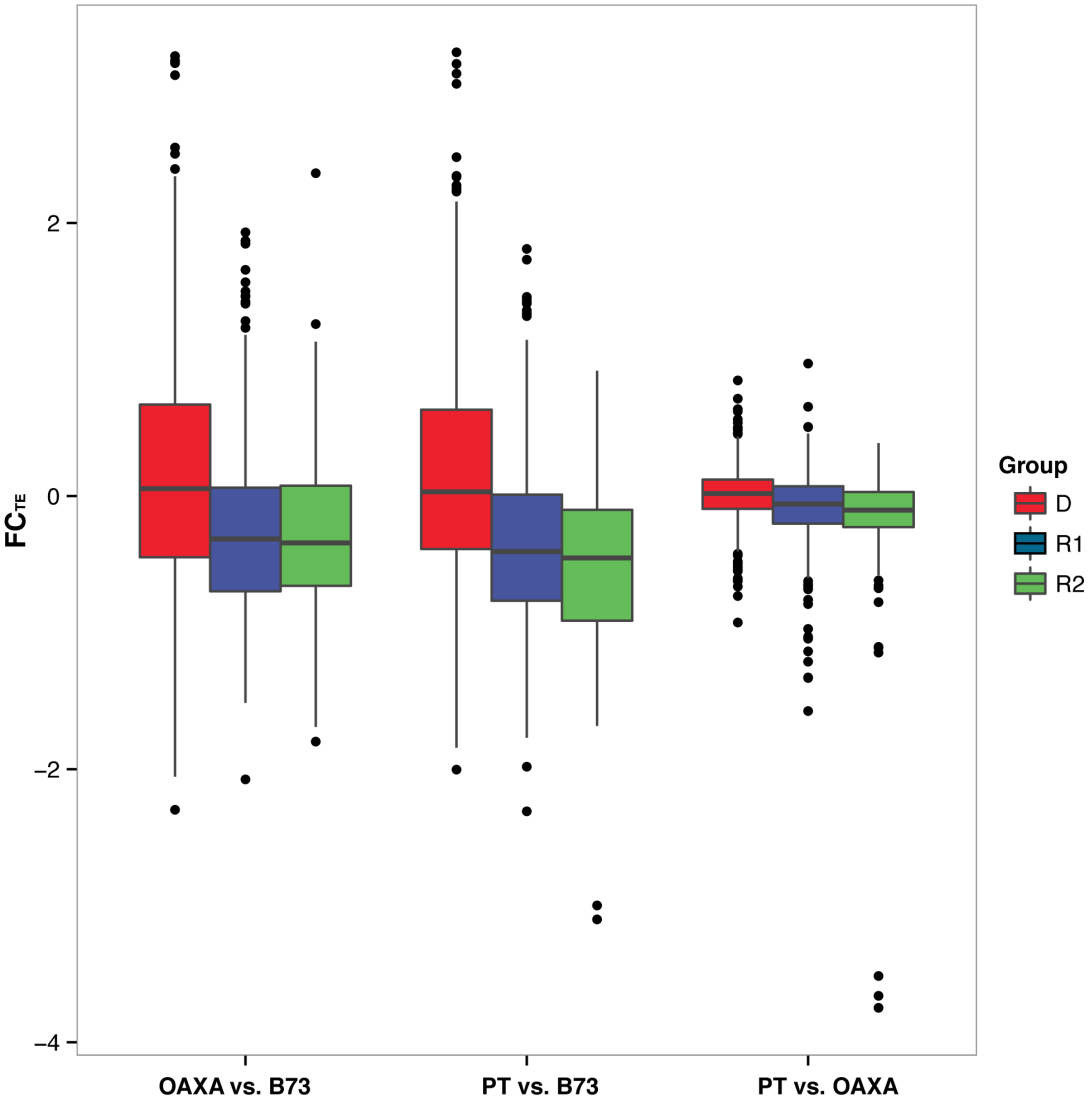
Boxplots of Fold Change in genomic reads for TEs (FC_TE_) within the D, R1 and R2 groups. The pairwise comparisons between accessions (B73, PT and OAXA) are indicated on the figure. The boxes indicate the first quartile (bottom line), the median (central line) and the third quartile (upper line). The whiskers represent the highest and lowest values of the data that are within 1.5 times the interquartile range of the box edges. The outliers are represented by dots.

Second, we assessed whether shifts in copy number were characteristic of the D, R1 and R2 groups. To address this issue, we measured the fold-change in abundance for each TE exemplar, or FC_TE_, as the log base 2 difference in normalized hits between two accessions (see Methods and Table S1). Note that FC_TE_ can be either positive or negative, representing increases in copy number for one or the other accession. We then plotted FC_TE_ values for each group and calculated the average FC_TE_ for each group (Table 3; Figure 5). In all pairwise comparisons between individuals, the average absolute value of FC_TE_ was higher for R1 and R2 than for DNA elements, differing significantly in all comparisons (p<<0.05, t-test). In contrast, the R1 and R2 groups did not differ consistently from one another in average FC_TE_ (*p = *0.017 for B73 vs. PT, but *p*>0.05 for the other pairwise comparisons; two-tailed t-test), suggesting that the two groups vary similarly in copy numbers between accessions. Thus, fold-change statistics suggest that the R1 and R2 groups varied in abundances more markedly among accessions than did the D group.

**Table 3:** Average Fold Change (FC) estimates for the three TE groups, based on pairwise comparisons between accessions.

Group	B73 vs. OAXA			B73 vs. PT			OAXA vs. PT		
	FC^1^_TE_	FC_22_	FC_24_	FC_TE_	FC_22_	FC_24_	FC_TE_	FC_22_	FC_24_
**D**	−0.156	0.1318	−0.1627	−0.164	−0.280	0.0066	−0.008	0.1484	0.1692
**R1**	0.264	0.6612	−0.0482	0.352	0.6877	0.2537	0.088	0.0264	0.3019
**R2**	0.330	−0.0976	0.0190	0.482	−0.275	0.0576	0.152	−0.1772	0.0385

1 FC is the average fold-change TE abundance and 22 nt and 24 nt siRNA across all of the TE subfamilies in each group. For each TE subfamily, the FC is the log 2 ratio of coverages between the two accessions, where coverage is #hits/#total hits.

### siRNA targeting does not correlate with copy number

**Figure 6:**
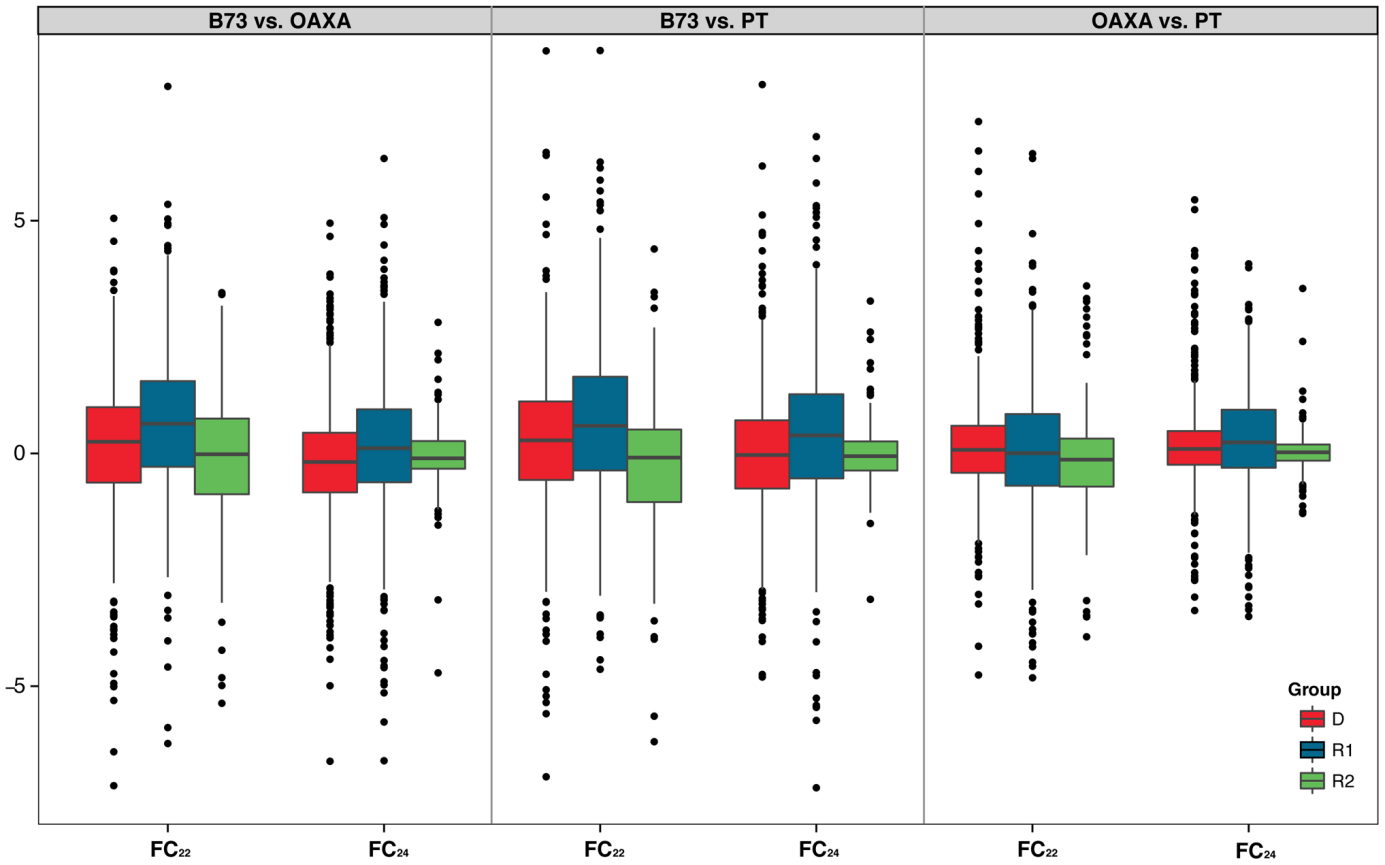
Boxplots of Fold Change in 22(FC_22_ and FC_24_) between accessions (B73, PT and OAXA), based on normalization by the upper quartile (Methods). The meaning of the boxes, whiskers and dots is defined in the legend of the Figure 4.

Because siRNA targeting is an important step in TE silencing and should therefore affect TE activity, we were interested in comparing copy number dynamics with the expression of small RNAs. That is, do copy number and small RNA expression covary? To address this question, we sequenced two siRNAs libraries from the same tissues (the third and fourth leaves) of PT and OAXA, resulting in >37.0×10^6^ 24 nt siRNAs and >15.0×10^6^ 22 nt siRNAs for each accession. We mapped siRNAs to the TEdb of 1526 elements, recorded the number of siRNA hits to each TE exemplar, and normalized expression by the upper quartile [31]. We calculated fold-change statistics for 22 nt (FC_22_) and 24 nt (FC_24_) siRNA for each TE subfamily in each of the three groups (Table S1). The results indicated that there were some marked differences in siRNA targeting for some individual D and R1 exemplars, with 222 and 174 subfamilies exhibiting absolute values of FC_22_ and FC_24_>2.0, respectively, in the B73 : PT comparison (Figure 6). However, the variability in FC for the R2 group was relatively small for both 22 nt and 24 nt siRNA expression (Figure 6).

The fold-change patterns based on TEs (Figure 5) and siRNAs (Figure 6) suggest both that siRNA targeting on R2 is highly conserved among accessions and that variation in siRNA expression is decoupled from TE copy number variation. We assessed this more formally using two approaches. The first was to assess the correlation between FC_TE_ vs. FC_22_ and between FC_TE_ vs. FC_24_ within groups or across all 1514 TE exemplars. No significant correlations were detected. For example in the B73:PT comparison, FC_TE_ was uncorrelated with FC_24_ (*r* ^2^ = 0.002; *p = *0.10) and FC_22_ (*r* ^2^ = 5×10^−6^; *p* = 0.94) across all of the TE exemplars in the R2 group. The second approach was to formulate and conduct statistical test of the hypothesis that TE copy number and siRNA expression change proportionally between individuals. We devised such a test (χ^2^ *_Prop_*) and applied it to all TE exemplars between accession pairs (see Methods and Text S1). Based on the χ^2^ *_Prop_* test, data from up to 917 TE subfamilies rejected the null hypothesis of proportionality between TE copy number (RPKM_TE_) and 24 nt siRNAs (Figure 4bc; Table 2). There were fewer rejections between TE copy number and 22 nt siRNAs, but up to 506 between B73 and PT. Thus, the overall pattern for our data is that, for any particular TE subfamily, the expression dynamics of siRNAs that target the TE do not closely mimic shifts in copy number, as measured by HTS data.

## Discussion

### The maize core genome

With the availability of genomic sequence data from multiple individuals, it has become possible to procure a snapshot of the “pan” (or whole) genome of a single species. The pan genome is defined to include a core component that is shared among individuals and also a non-core component that contains strain-specific DNA [21]. For maize, we know that the non-core component is substantial, because GS varies among individuals by up to at least 30% [20]. This and previous studies based on HTS genomic data suggest that the largest share of the non-core component is heterochromatic and knob repeats [18].

The core component is typified first by the genic fraction. For some of our analyses – i.e., those that employ χ^2^ *_Corr_* and χ^2^ *_Prop_* – we have assumed that the genic fraction represented by the Filtered Gene Set (FGS) is invariable among accessions. Under this assumption, the genic fraction provides an internal control for the ‘coverage’ of a library [17], [18]. We know that this is not a perfect assumption because the inbred lines B73 and Mo17 are estimated to vary in ∼180 annotated single copy genes and thousands of genes may differ between B73 and other germplasm [32]. It nonetheless seems reasonable to assume that the genic component is relatively static compared to either heterochromatic repeats or TEs.

TEs represent both the non-core and the core components of the pan-genome. They are part of the non-core component because they vary remarkably among maize individuals within a syntenous region [33], because the proportion of TEs within the genome varies among individuals [18] and because individual TE subfamilies vary in copy number between accessions (Table 2). However, we have also shown that the organization of TEs is core characteristic, in that TEs are conserved in three groups across a small but wide representation of maize germplasm. These three groups are class II DNA elements (D), low copy number class I RNA elements (R1), and a third set of higher copy RNA elements (R2). Recognition of this organization, and the consistency of this arrangement among maize genomes, is a novel contribution of this study.

### Fold-Change as a measure of shifts in TE abundance

To what extent to the three TE groups vary in copy number among accessions? We took two approaches to assess this question. The first was to compare estimated abundance changes for individual TEs (Figure 4a and Table 2). While we detect significant differences between accessions for many TE subfamilies, we urge caution in the interpretation of these results. For example, even though we have introduced an improved, modified and more conservative χ^2^ test, similar approaches are known to have high false positive rates despite the fact they are applied commonly to genomic data [e.g., 14]. This tendency is perhaps best illustrated by analyses of two biological replicates from reference [18] (Figure S3), for which we find significant differences in abundance for 331 TE subfamilies based on identical methods (χ^2^ *_corr_*; Table 2). This number provides a ‘baseline’ in which to evaluate our results. For our comparisons, the fewest significant differences were for 514 TE subfamilies between PT and OAXA (Table 2), suggesting that ∼200 ( = 514-331) TE subfamilies still differ in abundance between these accessions.

Our second approach was to report fold-change (FC_TE_) statistics that estimate shifts in abundance between accessions for groups of TEs. Our thinking is that FC_TE_ provides a better indication of overall trends by averaging across TE families, but this approach, too, is not without limits (Figure S3). That said, our analysis of FC_TE_ indicates that the R1 and R2 groups differ ∼1.3-fold in copy number on average between the B73 data and the data from the two landrace accessions (Figure 5). In contrast, the DNA elements vary little among accessions, but this may not be particularly surprising given their conservative mode of replication. FC_TE_ values also suggest that the B73 data differs more from the two landrace than the landraces differ from each other (Figure 4a), with the B73 data having a markedly higher abundance for R1 and R2 elements (Figure 5). At this point it is not possible to infer whether B73 is an outlier because of genetic differentiation (i.e., B73 is the outgroup to the two landraces) or because of a history unique to B73, such as inbreeding and intensive selection.

In this context, it is worth clarifying that FC_TE_ is designed to measure an outcome – i.e., differences in abundance – that likely summarize events across a range of mechanistic phenomena. On the one hand, transposition events contribute to differences in copy numbers between and among individuals, and hence FC_TE_ must encompass TE activity and transposition. However, FC_TE_ values may also reflect other processes that shift copy numbers, including phenomena like segmental duplication events, element deletion and natural selection, which likely differentially affects TE subfamilies that are located close to genes [34]. In fact, the Long Terminal Repeat (LTR) elements of the sort that constitute much of the R1 and R2 groups are particularly prone to deletion by unequal recombination [35], [36], and this process may be quite rapid. It is thus possible that element deletion contributes as much (or more) than transposition to FC_TE_.

Although FC_TE_ is not a direct measure of transposition events, it is not apparent that there are better measures to assess TE activity. For example, TE expression is often used as a measure of element activity, but TE transcription often does not reflect actual transposition events [13], [37]–[39]. There is, in fact, discordance between our estimates of abundance shifts between accessions (FC_TE;_ Figure 5) and expression within B73 (Figure 3ab). This discordance likely reflects that neither measure perfectly assesses transposition; TE expression is a poor measure of transposition activity but FC_TE_ measures an evolutionary outcome (abundance) rather than transposition directly.

### Little evidence that siRNA targeting covaries with copy number

A growing body of literature indicates that silencing mechanisms vary across TEs within the genome. For example, epigenetic modifications may be dependent or independent of siRNAs. The siRNA dependent processes may be, in turn, RDR2 dependent or independent, such as the silencing of MuDR elements by *mukiller* [40]. Even RDR2 mediated silencing seems to depend on a bevy of other characteristics, including the physical structure (nested or not) and chromosomal distribution of TEs [13], [29]; their copy number, length and age [22], [26], [34], [41], [42]; and their developmental timing [29], [39], [43]. While silencing varies among different TE families, we were interested in whether siRNA expression tracks copy numbers across individuals. We found no evidence that siRNA expression covaries with TE abundance, as shown by the lack of overall correlation between FC_TE_ and either FC_22_ or FC_24._ We also formulated explicit tests of proportionality (Table 2; Figure 4) that demonstrate that siRNA expression and TE abundance often do not covary. This low covariance is somewhat surprising: if shifts in TE abundance are due to element activity, it seems reasonable to assume that more siRNA is needed to silence more TE copies.

It is possible that our inferences about siRNA targeting are misled by our focus on leaf, as opposed to germline, tissue. To assess whether siRNA differs substantially among tissues, we reanalyzed siRNA data from previous publications [14], [44]. These data, which originated from B73 shoot apex and developing ear, were mapped to the TEdb, and then compared between tissues using the standard χ^2^ approach (χ^2^ *_Std_*). Similar to a previous study of methylation patterns [45], we find that the number of significant siRNA differences between tissues is smaller than that between individuals. We found that the number of TEs (of 1526 total) targeted differentially between tissues was 297 and 697 for 22 nt and 24 nt siRNAs, respectively. Notably, these differences may be inflated by the fact that the libraries used for these inter-tissue comparisons came from different growth conditions and even different experimental platforms [14], [44]. In contrast, ∼500 and ∼900 TE subfamilies are differentially targeted between B73 and the landrace accessions for 22 and 24 nt siRNAs (Table 2). Thus, while inter-tissue (or developmental) variation in siRNA targeting is considerable, it is less substantial than that between individuals, suggesting that the lack of covariance between TE abundance and siRNA expression may not be specific to leaf tissue.

### R1 and R2 have contrasting histories

Perhaps the most interesting aspect of this study is the previously unrecognized contrast between the R1 and R2 groups of retroelements. These groups consist of comparable Orders and Superfamilies of TEs (Table 1), and they exhibit similar levels of copy number variation among our sample of accessions (Figure 5). However, they differ in almost every other measurable characteristic, ranging from average length, to genomic context, to levels of siRNA targeting (Figures 1 & 6). They even vary as to whether methylation spreads to flanking regions from individual elements, because we have found that this is a phenomenon confined primarily to R2 elements [46] (data not shown). All of these descriptors suggest that the two groups have different dynamics with respect to the host response and also different evolutionary histories.

Given all of this information, the R2 group is still surrounded by at least two mysteries. The first is related to the observation that most R2 insertion occurred in a well-defined period, with little additional evidence of recent insertional activity (Figure 2). This observation suggests that these high-copy elements proliferated in a concerted burst of activity. Since the R2 group encompasses several TE families and Orders (Table 1), the event that triggered this burst must have had genome-wide effects. Yet the burst is too young to correspond to the ancient polyploid event in the maize lineage [47] and too old to correspond to maize domestication [48]; thus neither seem likely causes. The second mystery is why the age distribution signals little recent insertional activity despite copy number variation (Figure 5) and ongoing expression (albeit at a low level on a per-copy basis; Figure 3ab). If the age summaries are correct, we must conclude that: *i*) the tight variation of siRNA expression among individuals (Figure 6) reflects strong transpositional control on this group of elements, despite ongoing transcription and *ii*) measured variation in FC_TE_ between individuals reflect rearrangement and deletion events more than active transposition. Based on these considerations, our working hypothesis is that R2 elements are ‘mostly-dead’ (to paraphrase the 1987 movie ‘The Princess Bride’) with respect to ongoing proliferation via transposition.

While the R2 group is mysterious, the history of the R1 group is an even bigger puzzle. We initially hypothesized that these were relic elements, for two reasons. First, they have low copy numbers, which is indicative of limited replication. Second, the group is typified by a high proportion of RLX elements (Table 1), which have the features of class I retroelements but cannot easily be assigned to a particular family because they lack distinguishing structural features [22]. However, the bulk of evidence suggests that our hypothesis was wrong and that the R1 elements remain active. The evidence for this activity includes the fact that R1 elements are variable among individuals, as measured by FC_TE_ (Figure 5); are relatively highly expressed on a per-copy basis (Figure 3b); and are highly targeted by siRNAs relative to R2 elements (Figure 1e). Ongoing activity is also superficially supported by the age distribution of these elements (Figure 2), for which the mean age of insertion events is significantly lower than that of the R2 group and includes insertion times indicative of recent activity.

And yet, somewhat amazingly, 80% of TEs in the R1 group *decrease* in TE expression, by an average of −1.6 fold in shoot apical meristems, when the 24 nt siRNA biogenesis machinery is interrupted by a *mop1* mutation [13] (Figure 3c). At present, there is no clear explanation for this unexpected repression of expression, especially when one considers that R1 elements tend to be targeted by a high ratio of 24∶22 siRNAs (Figure 1e, Figure S2). One possibility is that R1 elements act as a generating source for siRNAs or other methylation signals [6], not unlike the piRNA loci of *Drosophila* or zombie elements hypothesized to serve as a source of siRNAs [49]. Under this scenario, their down-regulation in *mop1* would be consistent with an interruption of the host response mechanism. If this scenario were true, however, one would expect that the siRNAs that target group R1 TEs should cross-match TEs from other groups at higher than expected levels. We find that the highest percentage of different siRNA cross-matching occurred between R1-generated siRNAs and R2 TEs but at rates (∼2.0%) that seem too low to suggest that R1 elements act as a reservoir for the host response.

Altogether, our observations indicate that the R1 group is a heterogeneous set of elements that have been transpositionally active more recently than most R2 elements, perhaps for a longer period but at lower rates, as reflected by lower copy numbers. These observations suggest that the R1 group has been a long, slow, ongoing and active component of the maize pan-genome. In contrast, our evidence suggests the R2 group is ‘mostly dead’, under tight transpostional control and formed of a burst of ancient activity.

## Materials and Methods

### Sample preparation and library construction

#### Plant growth conditions

We analyzed two traditional maize cultivars, or landraces, called Palomero Toluqueño and Olote Colorado (a common variety of landrace Zapalote Chico), for which seeds were provided by CIMMYT, where the landraces are referenced as MEXI05 and OAXA522, respectively. We also included the reference maize inbred line B73, with seeds provided by the USDA-Agricultural Research Service (Ames, IA). Ten seeds per cultivar were planted in individual pots and grown in a growth chamber under controlled conditions of 12 h light at 26°C, 12 h dark at 20°C, a relative humidity of 70%, and 500–600 cal/cm2 of radiation per day. The third and fourth leaves of each plant were harvested when 12–13 cm long and then frozen in liquid nitrogen, and stored at −80 C. We chose to harvest these leaf tissues based on precedent in the literature [50] and the ease of establishing developmental homology.

#### Genomic and siRNA libraries

Leaf tissue from 10 different seedlings per landrace were pooled and ground in liquid nitrogen. Although the plants were not genetically identical, the distribution of genome sizes between the two landraces was not overlapping [20] and hence pooled samples give insights into average genomes of contrasting sizes. Genomic DNA was extracted from 1 g of pooled tissue using the Qiagen DNeasy plant mini kit. A paired-end library was built for each landrace using 1 µg of genomic DNA with the kit TruSeq Paired-End Cluster Kit v2.5 (Illumina PE-401-2510). Sequencing was performed in one lane on an IlluminaHiSeq 2000 sequencer. The genomic data are archived at NCBI Sequence Read Archive (SRA) under accession numbers SRX476038 (OAXA) and SRX476570 (PT). We also included genomic paired-end read data from B73 in our analyses [17] (SRA-SRP004910).

For all three accessions, total RNA was isolated from 1 g of pooled tissue using TRIzol reagent (Invitrogen) following the manufacturer's instructions. siRNA was extracted by running total RNA on a 15% PAGE gel and selecting bands in the 20 to 30 nt size range. Libraries for siRNAs were prepared from 100 ng of siRNA using the Illumina Truseq Small RNA Sample Prep Kit, according to the manufacturer's protocol. siRNA sequencing was performed in one lane on an IlluminaHiSeq 2000 sequencer per genotype using the Truseq SR cluster kit v. 2 for B73 and v.3 for PT and OAXA libraries. The siRNA data have been archived at the GEO database (GSE55730).

### Mapping procedures

#### Reference data sets

We mapped our genomic libraries to three reference databases: i) the filtered gene set (FGS) from RefGen_v2 (Release 5b.60) of the maize genome sequence [16]; ii) a custom-made database of knob and centromeric sequences (hereafter the KnobC database; Table S3) including 32 knob and 73 CentC maize sequences; and iii) the unique transposable element database (UTE) developed by Tenaillon et al. [17]. The siRNA libraries were also mapped against the FGS and the KnobC databases but also against the full TE exemplar database (TEdb) of 1526 elements [16].

#### Mapping genomic data

The pair-end datasets from PT, OAXA and B73 were mapped against all three reference sets separately. To map genomic reads to the UTE, we employed SSAHA2 version 0.1 [51] with default parameters, the “best” option and 80% homology, the criterion generally accepted as the level of similarity of reads within a single TE subfamily [25]. Only alignments >30 bp were counted, and each aligning read was counted as a “hit”. When multiple best-mapping reads were found for a single TE, we counted them as a single hit for that TE. The UTE virtually eliminates hits to multiple TEs, but reads that mapped to multiple TEs with the same score were discarded. The genomic data were mapped against the FGS and KnobC databases by the same procedure, except applying a 90% homology criterion for the FGS [17], [52].

For each accession, we recorded the total number of UTE, FGS and Knob hits. Because knob and centromeric sequences contain portions of TEs [53], we preferentially considered reads that mapped to both the Knob and UTE database as hits to the Knob database. We also considered reads mapping to both the UTE and FGS databases as TEs because the FGS may have not been filtered completely for the presence of TE-derived sequences [54]. Nonetheless, because there are few reads that map to more than one database, the overall results are robust to whether we preferentially mapped to Knob or the TE databases.

#### Mapping siRNA and RNAseq libraries

After sequencing siRNA, we trimmed adapters and 3′-end low quality nucleotides to ensure every read had three or more successive nucleotides with a quality score ≥20 at the 3′-end. Subsequently, we selected reads of 22 and 24 nt using CutAdapt [55]. These reads were filtered to eliminate rRNAs, rRNAs, miRNAs and snoRNAs and then mapped to the TEdb with bwa [56], using default settings. Uniquely and multiple mapped reads without mismatches were retained for further analyses. We divided the expression of reads with multiple targets by their number of targets.

We applied the same procedure to two small RNA libraries from developing ear and shoot apex tissues from [14], [44] (SRX143311, and SRX143309). We also analyzed RNAseq libraries from the transition leaf (from SRX172742 to SRX172747) and immature tassel (SRX172751 and SRX172752 from [57]) following the same protocol to trim adapters and to filter low quality nucleotides. Reads longer than 25 bp in length were mapped against the TEdb with bwa using default settings. Only uniquely mapped reads were considered for further analyses.

### Statistical analyses

#### Defining and comparing D, R1 and R2

To estimate an approximate TE copy number within a genome, we calculated RPKM [58] for each TE exemplar, as it is also described in [17]: 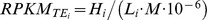, where 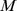 is the total number of reads mapped against the UTE, 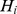 is the number of reads mapping to the 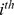 TE subfamily, and 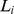 is the length in kilobases (kb) of the 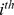 subfamily.

Before producing histograms comparing the number of subfamilies against their read coverage (Figure 1a) we removed families with RPKM <1.2 as corresponding to copy numbers <∼2 for B73. For the remaining TE subfamilies, we produced histograms and Rank-Frequency plots as an approximation of the Empirical Distribution Function (Figures 1ab and S2ab). We tested for differences among groups for several characteristics (length, copy number, etc.; Figures 1c–f and S2c–f). Because some of the variables did not fulfill the homogeneity of the variances required to apply linear models (Barlett's test, *p*<0.001; Shapiro–Wilk test, *p*<0.001), we applied non-parametric Kruskal-Wallis tests of significance.

#### Testing copy numbers between accessions

To compare TE copy numbers between accessions statistically, we used a standard χ^2^ (χ^2^ *_Std_*) consisting of a 2×2 table of observations, where two of the cells are the hits to the TE subfamily for both accessions and the other two cells are the hits to all other TE subfamilies for both accessions. From this table, expected values can be generated under the null hypothesis that the proportion of hits to the TE of interest is equivalent between accessions. In the case of one degree of freedom, as applied here, the χ^2^ is analytically identical to a Z-test, which is often used for testing differences in gene expression between RNAseq conditions. We applied the χ^2^ *_Std_* test to all TE subfamilies and corrected for experiment-wide error with a False Discovery Rate of *q*<0.001.

While commonly employed, this standard approach can generate an unacceptably high rate of false-positives if the genomic proportion of TEs varies substantially between accessions. We therefore devised a modified χ^2^ test (χ^2^ *_Corr_*). To generate an expectation under the null hypothesis that TE copy number is identical between accessions A and B, we assumed that the probability of a read falling in A is proportional to FGS coverage (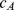) for A, with the same applying to accession B. Under this assumption, the expected number of hits to a particular TE subfamily, 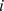, in genome A is, under the null hypothesis: 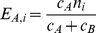 and similarly 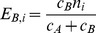, where 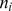 is the sum of observed hits 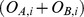. Defining 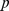 as 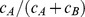, the χ^2^ used to test the difference between the observed values 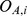 and 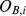 and their expectations, 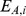 and 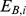, and it takes the form of a normal approximation to a binomial distribution:
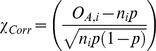
We applied χ^2^ *_Corr_*, based on the FGS coverage, to every TE subfamily separately, and then corrected for experiment-wide error with a False Discovery Rate of *q*<0.001.

#### FC computation

In order to compare among the different cultivars, we defined three variables, FC_TE_, FC_22_ and FC_24_. The three represent a base 2 logarithm of a quotient of normalized hits. For the genomic fold change, FC_TE_, the correcting procedure is simply to divide by the coverage of the DNA library, as determined by hits to the FGS, and to multiply by the average length of the reads. All TE subfamilies from the corresponding group were included in the computation, except those that have zero hits in some of the cultivars.

For the FC of siRNA expression, we normalized the reads by the value of the upper quartile, as recommended [31] before taking the base 2 logarithm of the ratio. For this analysis, we discarded TE subfamilies that had zero siRNA hits.

#### Deviations from proportionality

When both genomic and siRNA data are available, it is worth considering the null hypothesis of proportionality. In this case, the null hypothesis is a test of whether differences in siRNA targeting of a particular TE between accessions matches (or “covaries with”) differences in TE copy number. To perform this test, one needs to correct for the fact that the number of TEs may differ across accessions. Suppose that accessions A and B have different coverages for siRNA and genomic libraries. For a particular TE subfamily *i*, we first estimate the copy number from genomic DNA for a given accession as:
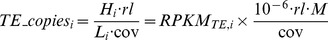
where 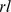 is the average read length in kb, 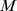 is the total number of reads mapped against the UTE, 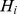 is the number of reads that map to the 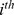 TE exemplar and 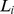, is the length of that TE subfamily in kb. 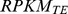 has been previously defined and takes into account the coverage of the DNA library, and 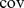 is the coverage of the siRNA library.

Given an estimate of copy number, our null hypothesis of proportionality is that ratio of the copy number of the TE representing subfamily 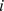 (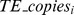) and its coverage by targeting siRNA (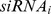) is equivalent between accession A and B. That is, H_O_:
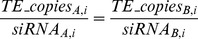
To test this hypothesis requires estimation of a number of parameters, including the (unknown) global coverage of the siRNA libraries from accessions A and B; these values are necessary to generate the expected values for inclusion in a χ^2^ (χ^2^ *_Prop_*). We include a full derivation of the approach in the Supplementary Text.

## Supporting Information

Figure S1 Characterization of the subset of 340 TE exemplar subfamilies that exhibited differential expression in the *mop1* mutant [13] after separation into the three TE groups. Left, their length; middle, their abundance (RPKM_TE_); right; their 24∶22 nt siRNA-targeting ratio.
(PDF)

Figure S2 Figures analogous to Figure 1 for OAXA (a–f) and PT (g–l) data.
(PDF)

Figure S3 Outcome of FC_TE_ analyses of replicated samples of B73 (SSR447984 and SSR447986) from [18]. The boxes indicate the first quartile (bottom line), the median (central line) and the third quartile (upper line). The whiskers represent the highest and lowest values of the data that are within 1.5 times the interquartile range of the box edges. The outliers are represented by dots. Because these are replicated samples, the expectation of FC_TE_ for each group is zero. As expected, the mean values for the R1 and R2 groups are centered on zero. FC_TE_ for the D group exhibits more variability, but zero is nonetheless captured within the first and third quartiles.
(PDF)

Table S1 Characterization of the exemplar TE subfamilies - including the observed hits based on genomic reads, 22nt siRNAs, 24 nt siRNAs and their fold changes (FCs) – for all three accessions (B73, OAXA and PT).
(XLSX)

Table S2 Information about the subset of 340 TE subfamilies assessed between the *mop1* mutant and the wild type.
(XLSX)

Table S3 The Genbank references of the sequences in the Knob and CentC reference database.
(XLSX)

Text S1 Derivation of the test of proportionality, which tests the null hypothesis, for any single TE exemplar, that the ratio of TE copy number to siRNA targeting is equivalent between two accessions.
(PDF)
