# Supplementary figures and images for: Three Groups of Transposable Elements with Contrasting Copy Number Dynamics and Host Responses in the Maize (Zea mays ssp. mays) Genome
Source: PLoS Genet. 2014 Apr 17;10(4):e1004298. doi: 10.1371/journal.pgen.1004298 (PMC3990487; doi:10.1371/journal.pgen.1004298)

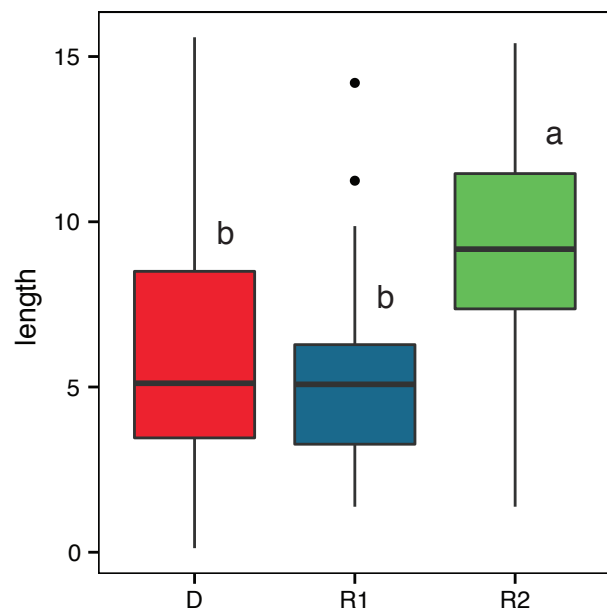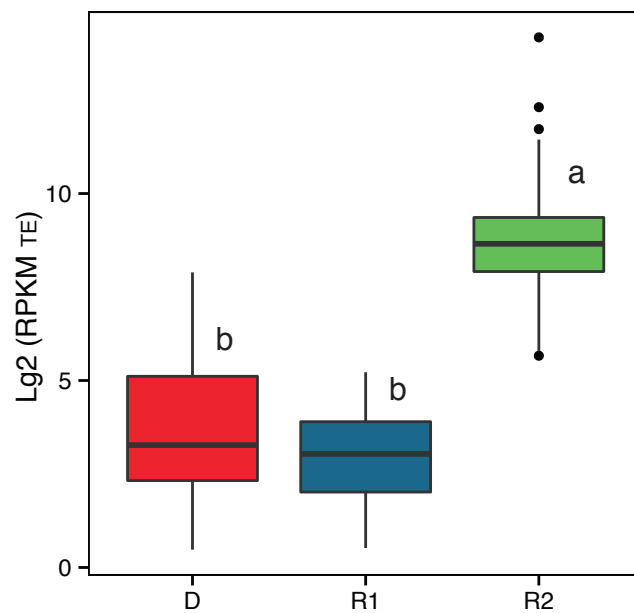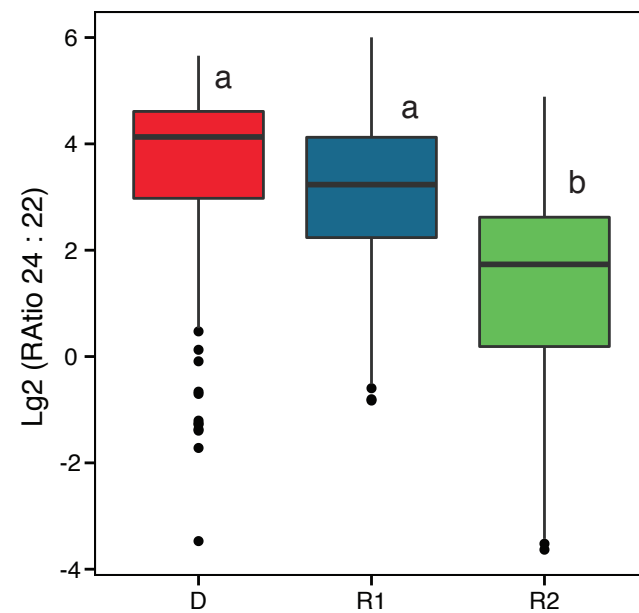

Supplement: Figure S1 — Characterization of the subset of 340 TE exemplar subfamilies that exhibited differential expression in the mop1 mutant [13] after separation into the three TE groups. Left, their length; middle, their abundance (RPKMTE); right; their 24∶22 nt siRNA-targeting ratio. (PDF) [file pgen.1004298.s001.pdf]

**Figure 2S: OAXA**

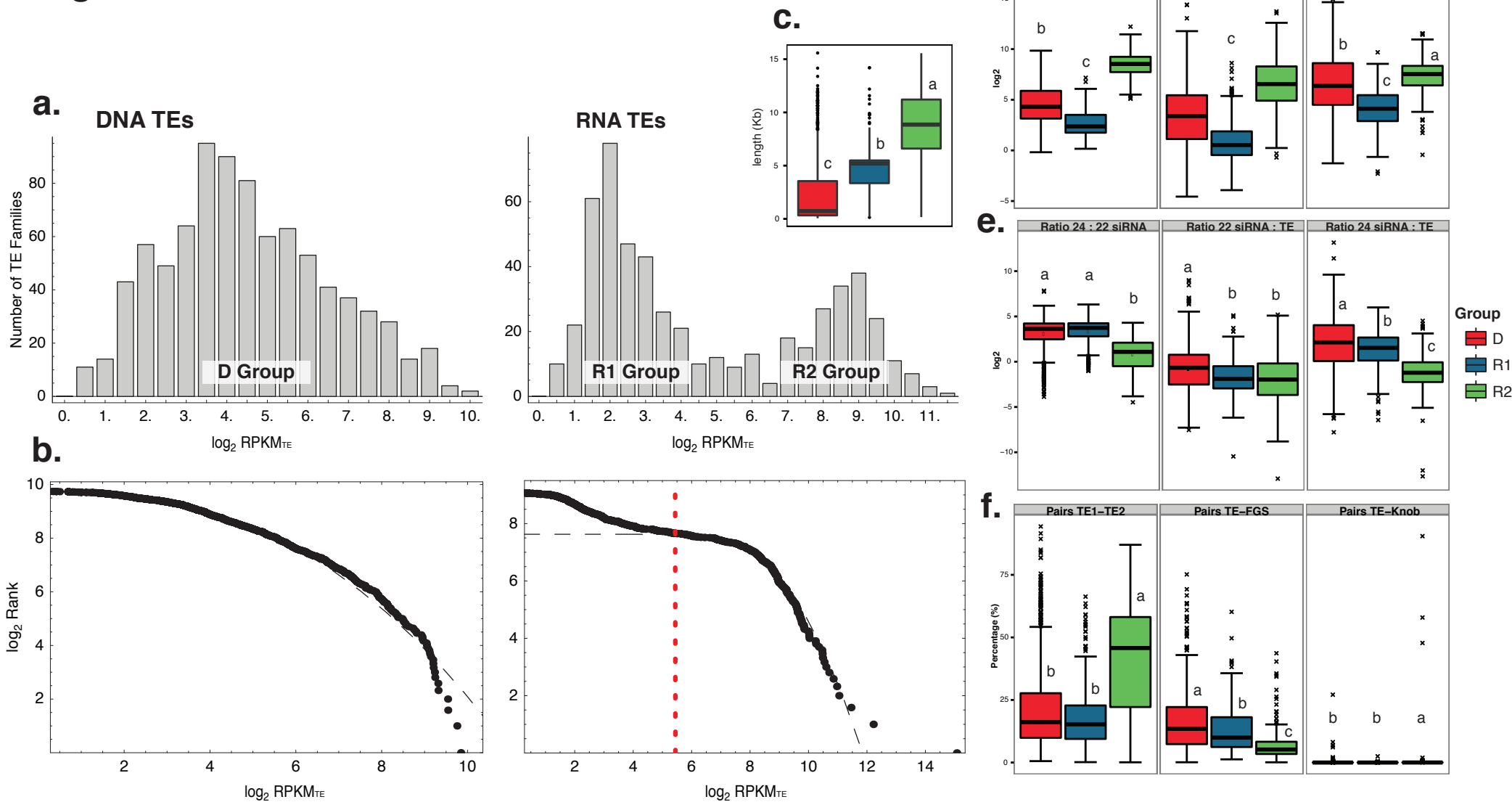

Figure 2S: PT

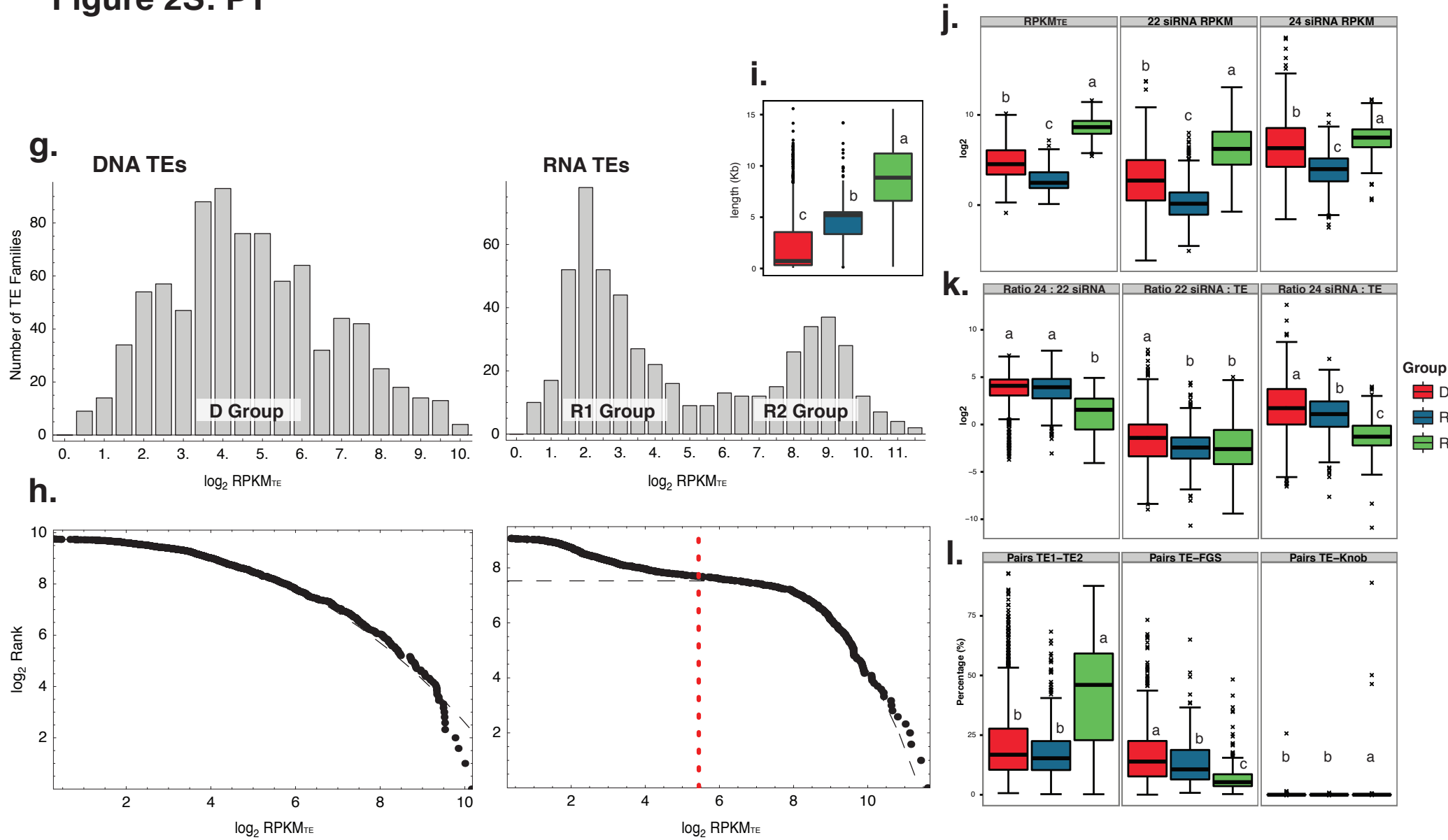

Supplement: Figure S2 — Figures analogous to Figure 1 for OAXA (a–f) and PT (g–l) data. (PDF) [file pgen.1004298.s002.pdf]

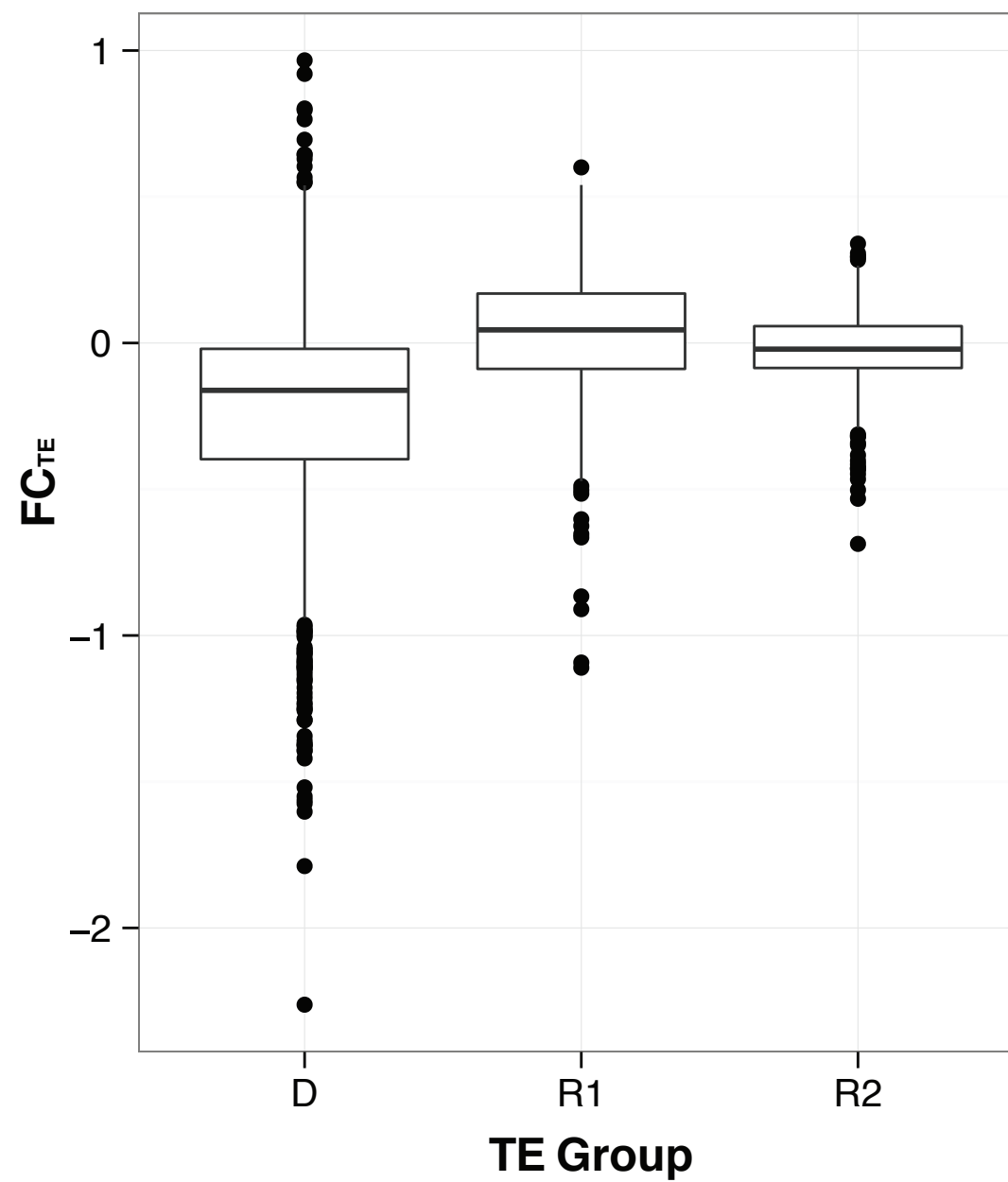

Supplement: Figure S3 — Outcome of FCTE analyses of replicated samples of B73 (SSR447984 and SSR447986) from [18]. The boxes indicate the first quartile (bottom line), the median (central line) and the third quartile (upper line). The whiskers represent the highest and lowest values of the data that are within 1.5 times the interquartile range of the box edges. The outliers are represented by dots. Because these are replicated samples, the expectation of FCTE for each group is zero. As expected, the mean values for the R1 and R2 groups are centered on zero. FCTE for the D group exhibits more variability, but zero is nonetheless captured within the first and third quartiles. (PDF) [file pgen.1004298.s003.pdf]
