# Supplementary material for: Three Groups of Transposable Elements with Contrasting Copy Number Dynamics and Host Responses in the Maize (Zea mays ssp. mays) Genome
Source: PLoS Genet. 2014 Apr 17;10(4):e1004298. doi: 10.1371/journal.pgen.1004298 (PMC3990487; doi:10.1371/journal.pgen.1004298)
Supplement: Text S1 — Derivation of the test of proportionality, which tests the null hypothesis, for any single TE exemplar, that the ratio of TE copy number to siRNA targeting is equivalent between two accessions. (PDF) [file pgen.1004298.s007.pdf]

Comparison of siRNA Targeting Among Cultivars Weighted by $RPKM_{TE}$ and Coverage

1 Preliminaries

When comparing the amount of siRNA that hits on a certain TE in two different cultivars, care must be taken to account for the different number of copies of the same TE. The relevant variable should be the siRNA hits over the number of copies, a measure of which could be the associated RPKM over the coverage.

Also, to compare count-like data by using a χ^2 test it is important not to rescale the count number, but the expected number of occurrences of an event (i.e. hits of siRNA on a given TE subfamily) should nevertheless reflect the fact that the number of TEs is different across cultivars.

Before continuing, we need to establish the definition of the RPKM and its relations with the number of copies. First we start with the RPKM of the ith TE subfamily:

$$RPKM_{TE,i} = \frac{H_i}{L_i \cdot M \cdot 10^{-6}}, \tag{1}$$

where H_i is the number of reads mapping to the ith subfamily, L_i is its length in kb and M is the total number of mapped reads against the UTE.

An estimation of the number of copies from the number of mapped reads could be

$$Copies_i = \frac{H_i \cdot rl}{L_i \cdot \text{cov}}, \tag{2}$$

where rl the length of the reads in kb and cov is the estimated coverage of the library.

Equivalently one could write the estimated copy number in terms of the $RPKM_{TE}$:

$$Copies_i = RPKM_{TE,i} \frac{10^{-6} \cdot rl \cdot M}{\text{cov}}. \tag{3}$$

Here is a table with a summary of the relevant statistics of each cultivar:

Cultivar	cov	Total Reads	Length of the Reads (bp)
B73	1.32	23056452	84/104
P. Toluqueño	5.9	66688407	100/100
Oaxaca	4.6	59751218	100/100

2 Methodology

Suppose that two given cultivars, A and B, have a different coverage of the RNA library and the DNA library. Now, assume that $x_{A,i}$ and $x_{B,i}$ are the expected number of siRNA hits on the ith TE subfamily for cultivar A and B respectively. Then, our null hypothesis states that:

$$\frac{x_{A,i}}{x_{B,i}} = \frac{Copies_{A,i}}{Copies_{B,i}} \frac{covRNA_A}{covRNA_B}, \tag{4}$$

that is to say, the number of hits is proportional to the number of copies of the TEs and to the (unknown) coverage of the siRNA library. Note the way in which we estimate the number of copies involves a coverage of the DNA library, see previous section. Below we describe a way to estimate the ratio of the coverages of the siRNA libraries, an unknown constant independent of the TE subfamiliy.

For each TE, in order to find an expected number of instances, we have to take into account the constraint:

$$x_{A,i} + x_{B,i} = S_i, \tag{5}$$

where S_i is the total number of siRNA hits across cultivars.

The expected values of $x_{A,i}$ and $x_{B,i}$ can be computed from the previous equations, which results in a simple lever rule:

$$\begin{aligned} x_{A,i} &= S_i \frac{covRNA_A \, Copies_{A,i}}{covRNA_A \, Copies_A + covRNA_B \, Copies_B} \\ x_{B,i} &= S_i \frac{covRNA_B \, Copies_{B,i}}{covRNA_A \, Copies_A + covRNA_B \, Copies_B} \end{aligned} \tag{6}$$

Now, in order to estimate the value of the ratio $covRNA_A/covRNA_B$ we make use of an additional constraint, which is that the sum of all the $x_{A,i}$ has to be equal to the total number of mapped siRNA hits on the transposons for cultivar A, S_A:

$$\sum_{i=1}^{N} x_{A,i} = S_A, \tag{7}$$

where N is the number of different TEs.

Taking into account all the previous equation we obtain the following equation for the ratio $covRNA_A/covRNA_B$:

$$\sum_{i=1}^{N} S_i \frac{\frac{covRNA_A}{covRNA_B} Copies_{A,i}}{\frac{covRNA_A}{covRNA_B} Copies_{A,i} + Copies_{B,i}} = S_A. \tag{8}$$

There is an equivalent equation for cultivar B, but it is easy to prove that it gives the same solution, i.e. it is not independent.

The previous equation is easy to solve numerically, which allows us to find the ratio of the coverages of the RNA libraries and hence the expected values, by using Eq. (6).

Once we have the values of the expected values of the cultivars, we can find the χ^2 statistic in the usual way, i.e.

$$\chi^2_{Prop,i} = \frac{(x_{A,i} - \tilde{x}_{A,i})^2}{x_{A,i}} + \frac{(x_{B,i} - \tilde{x}_{B,i})^2}{x_{B,i}}, \tag{9}$$

where the tildes denote the observed variables. Since the sum of $x_{A,i} + x_{B,i}$ is constrained, there is only one degree of freedom.
